# Nurses the Exclusive Occupiers of a New Field

**Published:** 1919-08-30

**Authors:** 


### MINISTERS OF HEALTH.

### Nurses the Exclusive Occupiers of a New Field.

During the past three weeks I have been endeavouring to set out for the consideration of the nursing profession the prizes which the work of national reconstruction places within reaching distance, and the question at issue is—Will the members respond?

### An Intermediary Rank.

I desire to be not only clearly, but unmistakably understood. The requirements of the Health Ministry make it possible to establish a new and intermediary rank between the trained nurse and the medical doctor. The health visitor will receive a more extended training than the nurse, her emoluments will be higher, and her opportunities for initiative will be greater. It is not possible at this stage accurately or even approximately to define her outlook, but it includes very much more than appears from any printed regulation, or from any contemplated regulation. The Minister of Health himself could not if he would prepare a programme setting forth the duties and the prospects of women workers who may take service under his Department.

### The Vast and Unexplored.

The field is vast and unexplored. The demand is enormously in excess of the supply, and the work is fascinatingly interesting in addition to its being patriotic and humanitarian. There is a theory in economics that the demand creates, or, at all events, precedes, a supply. This may be true, but if so it is only a fraction of the whole truth. Ask any fashionable milliner or robe-maker about supply and demand. You will learn that it is the business of the trade to create both, and that the supply usually comes first. Make something useful or ornamental, and immediately everybody wants it. The common safety-pin is a simple illustration. This was not invented or made because there was a public demand. The demand followed. Produce your able, accomplished, and zealous health visitor, and the world will wonder how it happened that so indispensable a worker was not long ago discovered. Set her a task, and a year hence she will claim a higher and more difficult one. She will become as necessary as the telephone, and the calls for her services will for years be far more numerous than can be answered. Who shall set a boundary to the triumphant march of the woman worker?

### The Nurse's Heritage.

I think I have made it quite clear that if they choose to do so, nurses can become the exclusive, or the almost exclusive, occupiers of the new field. The Minister of Health has thrown the doors open to the world, but he has expressed a marked preference. The most competent potential health visitors, he says, are specially trained nurses and midwives. But the fact remains that there is none so specially trained, and, like the man in the parable, if the proper guests fail to arrive he must go out into the highways and the byways, for his house must be filled. It is extremely important that the nurse should realise that this particular field is her rightful heritage, and that the other people are the cuckoos. I am afraid that it will take some time and trouble to make this fact thoroughly understood and appreciated. The reason for this is because it appears to appeal only to the coming generation. The special training that is essential is outside the reach of a very large number of trained nurses. They cannot afford to leave their posts for a year, which is the shortened period of training. A great many of the younger and more enterprising will doubtless appreciate the opportunity and take advantage thereof. But it is above all to probationers, present and to become, that the slogan calls, and it is hard to find champions for what I may almost describe as posterity. Only the bigger souled women of the profession, who love it for its traditions, for what it stands for in the world, and to whom a lowering of the standard would be a cause of pain—only such as those can bring about the reform that will ensure the nurse being the heir to her estates.

### The Whole Profession Will Benefit.

In reality, however, the whole profession will benefit by the opening up of a new field of employment. With a growing demand for workers, salaries automatically increase, and, as I have more than once observed, an improvement at the lower end of the ladder is bound to affect those who occupy the higher rungs. It does not follow if a matron's salary is increased that the ward sister's pay rises. But begin below, and corresponding benefit all along the line is inevitable. And if what I have been urging is to become more than an academic discussion the nurses' leaders will have to swing out into mid-stream and be active. They will have to convince hospital committees that by making a radical change their institutions will be recruited from high-class sources.

### The Ministry of Health.

Candidates for appointments under the Health Ministry will find it to their advantage to graduate in the training-schools, for it is made abundantly clear that in all cases the qualified nurse will receive preference when vacancies are being filled. But there must be no tinkering. None but girls who have received a good education will be a success, no matter how experienced or how highly trained they may afterwards become in technical matters. The probationer must be in the true sense a student. She must have time for study, class work, and recreation, and her long apprenticeship of three years must not be spent, as hitherto, almost constantly at the bedside or at other exacting duties. She must be permitted to live the normal life of a healthy, intelligent, and ambitious young woman.

IERNE.

